# A271 EFFECT OF ANTI-TNF AGENTS ON DNA METHYLATION IN PERIPHERAL BLOOD OF PATIENTS WITH INFLAMMATORY BOWEL DISEASE

**DOI:** 10.1093/jcag/gwad061.271

**Published:** 2024-02-14

**Authors:** J Venner, J Francis, J Rumore, M Sargent, M Jones, C Bernstein

**Affiliations:** Gastroenterology, University of Manitoba, Winnipeg, MB, Canada; Gastroenterology, University of Manitoba, Winnipeg, MB, Canada; Gastroenterology, University of Manitoba, Winnipeg, MB, Canada; Gastroenterology, University of Manitoba, Winnipeg, MB, Canada; Gastroenterology, University of Manitoba, Winnipeg, MB, Canada; Gastroenterology, University of Manitoba, Winnipeg, MB, Canada

## Abstract

**Background:**

Despite the success and ongoing use of tumor necrosis factor inhibitors (TNFi) in the treatment of inflammatory bowel disease (IBD), there are no predictors of response to therapy. While much research has gone into understanding genetic risks for IBD, less work has been done exploring epigenetic associations with treatment. Hence, we wondered whether epigenetic changes were associated with failure of TNFi in IBD.

**Aims:**

Describe the association of TNFi response with DNA methylation in patients with IBD.

**Methods:**

Participants ≥18 years with IBD (N=169) were selected from the Inflammation, Microbiome, and Alimentation: Gastro-Intestinal and Neuropsychiatric Effects (IMAGINE) Strategy for Patient Oriented Research (SPOR) Network. At enrollment participants completed a questionnaire that included their disease diagnosis, therapy, smoking history, and disease activity using the validated IBD Symptom Inventory (IBDSI). Patients provided whole blood samples that were processed for and run on Illumina DNA methylation arrays.

**Results:**

The 169 patients were in three treatment groups: TNFi naïve (N=98, never exposed to an anti-TNF agent), TNFi responder (N=32, inactive disease on anti-TNF agent), and TNFi nonresponder (N=39, active disease not on any biologic at time of enrollment). The mean symptom score (SIBDSI) was different across the three groups (pampersand:003C0.001): TNFi nonresponders having a higher SIBDSI (24 ± 6.1) than the TNFi naïve (9.1 ± 5.5) or responder (8.3 ± 4.3) treatment groups. Relative proportions of leukocyte populations were estimated using DNA methylation. CD4 and CD8 T cell and B cell counts were higher (pampersand:003C0.05) in the nonresponders and responders versus TNFi naïve group. Neutrophil counts were lower (pampersand:003C0.05) in the TNFi nonresponders and responders compared to the TNFi naïve group (Figure 1). There was a trend towards increased epigenetic estimates of age acceleration (pampersand:003E0.05) in nonresponders versus responders and TNFi naïve patients, likely driven by disease activity (data not shown). Epigenome-wide analysis of the three groups revealed 16 CpGs for responder versus naïve (e.g. CDK5 regulatory subunit-associated protein 1-like 1 (CDKAL1), and two CpGs for nonresponders versus responders (Table 1). This includes a CpG for guanine nucleotide-binding protein subunit gamma-2 (GNG2) that was shared between the two comparisons.

**Conclusions:**

Exposure to TNFi is associated with changes in peripheral leukocyte populations independent of response to treatment, indicating a TNFi effect. This implies that anti-TNF treatment is having some effect on patients even if there is clinical nonresponse. Differentially expressed CpGs implicates possible markers of response to treatment, particularly CDKAL1, a gene that has been associated with TNFi response in psoriasis.

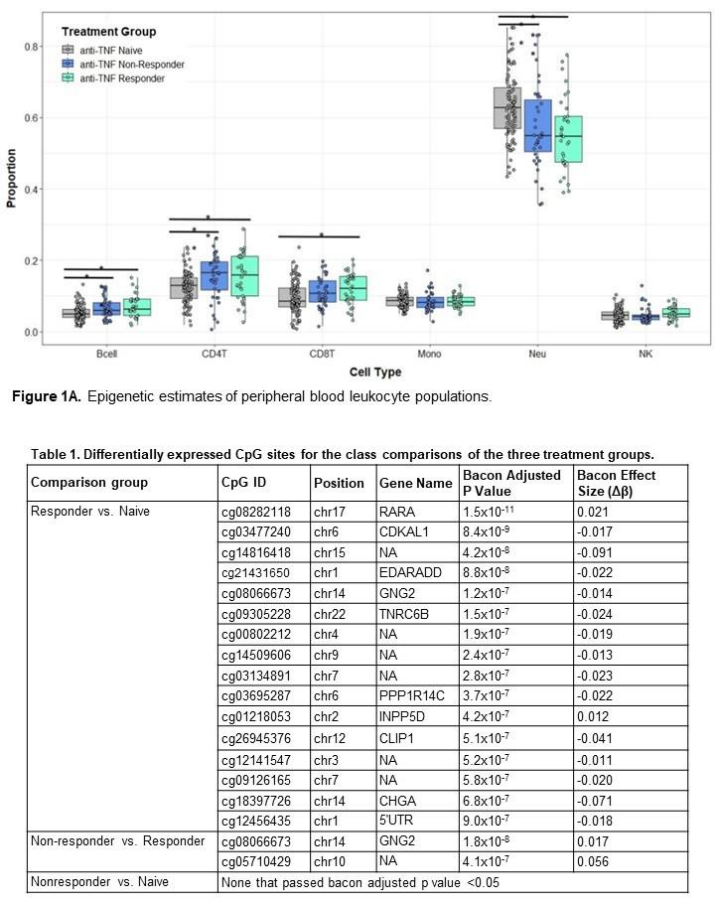

**Funding Agencies:**

Guts and Roses Charity

